# Case Report: A Novel *GJB2* Missense Variant Inherited From the Low-Level Mosaic Mother in a Chinese Female With Palmoplantar Keratoderma With Deafness

**DOI:** 10.3389/fgene.2022.938639

**Published:** 2022-07-22

**Authors:** Xinyuan Tian, Chuan Zhang, Bingbo Zhou, Xue Chen, Xuan Feng, Lei Zheng, Yupei Wang, Shengju Hao, Ling Hui

**Affiliations:** ^1^ School of Public Health, Gansu University of Chinese Medicine, Lanzhou, China; ^2^ Center for Medical Genetics, Gansu Provincial Maternity and Child Health Hospital, Gansu Provincial Clinical Research Center for Birth Defects and Rare Diseases, Lanzhou, China

**Keywords:** *GJB2* gene, palmoplantar keratoderma, hearing loss, dominant variant, mosaicism

## Abstract

Dominant variants in the gap junction beta-2 (*GJB2*) gene may lead to various degrees of syndromic hearing loss (SHL) which is manifest as sensorineural hearing impairment and hyperproliferative epidermal disorders, including palmoplantar keratoderma with deafness (PPKDFN). So far, only a few *GJB2* dominant variants causing PPKDFN have been discovered. Through the whole-exome sequencing (WES), a Chinese female patient with severe palmoplantar hyperkeratosis and delayed-onset hearing loss has been identified. She had a novel heterozygous variant, c.224G>C (p.R75P), in the *GJB2* gene, which was unreported previously. The proband’s mother who had a mild phenotype was suggested the possibility of mosaicism by WES (∼120×), and the ultra-deep targeted sequencing (∼20,000×) was used for detecting low-level mosaic variants which provided accurate recurrence-risk estimates and genetic counseling. In addition, the analysis of protein structure indicated that the structural stability and permeability of the connexin 26 (Cx26) gap junction channel may be disrupted by the p.R75P variant. Through retrospective analysis, it is detected that the junction of extracellular region-1 (EC1) and transmembrane region-2 (TM2) is a variant hotspot for PPKDFN, such as p.R75. Our report reflects the important and effective diagnostic role of WES in PPKDFN and low-level mosaicism, expands the spectrum of the *GJB2* variant, and furthermore provides strong proof about the relevance between the p.R75P variant in *GJB2* and PPKDFN.

## Introduction

Hearing loss (HL) is a common genetic disorder, involving a multitude of different genes and showing different inheritance patterns. A few forms of HL are related to variants in connexins, especially connexin 26 (Cx26, NP_003995.2) coded by the gap junction beta-2 (*GJB2*) gene. The rapid intercellular communication is mediated by gap junction channels polymerized from connexins, which are small transmembrane proteins. Variants in the *GJB2* gene can lead to hearing loss with or without skin abnormalities (Lee et al., 2010).

Currently, over 400 different variants have been discovered in the *GJB2* gene, the predominance of which causes non-syndromic autosomal recessive 1A deafness (DFNB1A), such as p.M34T, p.V37I, p.V84L, p.T86R, p.L90P, and p.R143W. Only a few *GJB2* heterozygous variants are associated with autosomal dominant 3A deafness (DFNA3A) and various syndromic hearing loss (SHL) conditions, such as Vohwinkel syndrome (VOWNKL), keratitis–ichthyosis–deafness (KID) syndrome, ichthyosis hystrix-like deafness (HID) syndrome, Bart–Pumphrey syndrome (BAPS), and palmoplantar keratoderma with deafness (PPKDFN, OMIM#148350) (Xu and Nicholson, 2013).

PPKDFN is an autosomal dominant syndromic hearing loss caused by heterozygous variants in the *GJB2* gene on chromosome 13q12.11. The genetic disease can cause diffuse or localized hyperkeratosis of the palms and soles and hearing impairment, and most of them are congenital. To date, the following variants in *GJB2* have been identified in PPKDFN: p.K22N, p.M34K, p.E42del, p.N54H, p.G59A, p.G59R, p.H73R, p.R75W, p.R75Q, p.G130V, p.S183F, and p.R184Q. Two of the most common variants are p.R75W and p.R75Q. In this study, we report a Chinese patient with PPKDFN caused by a novel p.R75P heterozygous variant in the *GJB2* gene, which was inherited from her mother who exhibited somatic mosaicism that was detected by ultra-deep targeted sequencing.

## Case Description

The proband and her parents underwent genetic testing at the Medical Genetics Center of Gansu Provincial Maternity and Child Health Hospital. The proband is a 24-year-old female who experiences severe hearing loss and mute since she was 2 years old. At the same time, she has severe symptoms of palmoplantar hyperkeratosis, whose palms and soles are rough and prone to peeling and cracking. Her mother also has mild keratoderma of the palms and soles. Due to personal privacy, the proband refused to provide photographs of the relevant phenotypes. This study was approved by the local Ethics Committee and conducted according to the tenets of the Declaration of Helsinki.

## Methods

### Whole-Exome Sequencing

DNA was obtained from the peripheral blood of the patient and her parents using TIANGEN TIANamp Genomic DNA Kit, and the purity and concentration of DNA were determined using a NanoDrop 2000 nucleic acid quantifier (DNA concentration was controlled at 50∼250 ng/μl). DNA was submitted to Chigene Co., Ltd. for trio whole-exome sequencing (trio WES). To target the 39-Mb protein-coding region (19,396 genes) of the human genome and cover 51 Mb of end-to-end tiled probe space, the exome was captured from peripheral blood DNA using xGen Exome Research Panel v2.0 (IDT, IA, United States) that consists of 429,826 individually synthesized and quality-controlled probes. Paired-end sequencing was performed to sequence not less than 99% of the target sequence using the Illumina technology platform (Santa Clara, CA, United States).

### Sanger Sequencing

Primers were designed by online primer design software Primer 3 to cover the *GJB2* (NM_ 004004.6) gene exon 2 and its flanking sequences (forward primer sequence: CAT​GCT​TGC​TTA​CCC​AGA​CTC​A; reverse primer sequence: TAG​CGA​CTG​AGC​CTT​GAC​AGC). PCR reaction system and conditions: 2×PCR Mix 12.5 μl, ddH_2_O 10 μl, forward and reverse primers 0.5 μl each, and DNA 1.5 μl; denaturation at 95°C for 5 min, then denaturation at 94°C for 30s, annealing at 62°C for 30s, extension at 72°C for 1 min, 20 cycles were performed, followed by denaturation at 94°C for 30 s, annealing at 58°C for 30 s, extension at 72°C for 1 min, and extension at 72°C for 10 min after 15 cycles. The PCR products were gel-purified on a 1.0% agarose gel and used for sequencing using BigDye Terminator. Finally, capillary electrophoresis was performed on the ABI 3500DX genetic analyzer, and the Sanger sequencing results and the *GJB2* gene reference sequence were compared and analyzed using SeqMan.

### Ultra-Deep Targeted Sequencing

DNA was extracted from peripheral blood of the probands mother, and a unique molecular tag was added to each original DNA fragment. After two rounds of PCR amplification, a sequencing library of the target site was constructed. The PCR amplification products were purified by magnetic beads and subjected to high-throughput sequencing using the DNBSEQ-T7 platform of the NovaSeq series. DNA templates from different sources can be distinguished according to different tag sequences, and real variants can be distinguished, thereby improving the detection sensitivity and specificity. Burrows–Wheeler Aligner (Li and Durbin, 2010) was used to compare the raw data with the reference sequence, the hg19 genome. Self-developed analysis software was used to screen targeted SNP and Indel 1∼30bp in length. The reliability of variants was assessed by the ratio of mutated reads and depth between the controls and the subjects.

### *In Silico* Analysis

The protein domain model diagram was drawn utilizing Illustrator for Biological Sequences v1.0 (IBS) (W. Liu et al., 2015). The 3D structural analysis of the GJB2 protein was carried out using PyMOL (Rigsby and Parker, 2016). Protein morphology and sequence characteristics were illustrated by Protter (Omasits et al., 2014).

## Results

**FIGURE 1:**
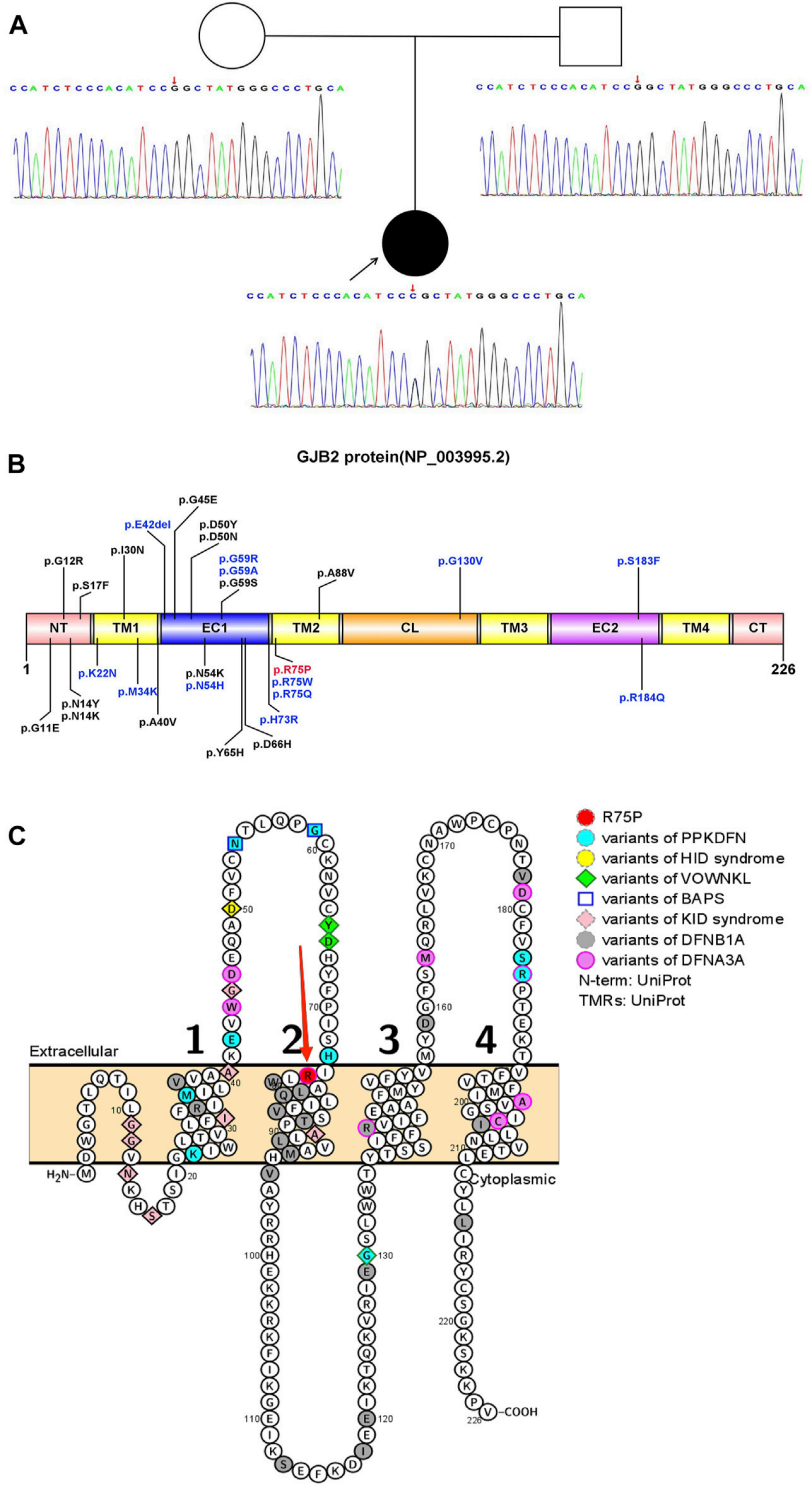
Pattern diagram of the GJB2 protein domain, protein functional region, and transmembrane domain analysis. **(A)** Pedigree and Sanger sequence of *GJB2* c.224 in the proband and parents. A novel heterozygous missense variant in *GJB2* c.224G>C was detected in the proband suffering from PPKDFN, but neither the father nor the mother of the proband was detected for the same. **(B)** Currently reported variant sites that cause PPKDFN (blue font) and other SHL (black font), and the variant site in this study (red font). **(C)** Locations of reported pathogenic *GJB2* variants (variant positions in different diseases are represented by different colors and shapes). Labels of TMs are represented by 1, 2, 3, and 4 (black numbers).The p.R75P variant site (red arrow) is at the junction of EC1 and TM2.

From the proband, we detected a heterozygous missense variant, c.224G>C (p.Arg75Pro, p.R75P) in the *GJB2* gene (Figure 1A), with the heterozygous ratio of 84/172. The variant was present in a frequency of 2/83 of the reads in the mother, suggesting the possibility of mosaicism in the mother. According to ACMG guidelines, the variant was assessed as pathogenic (PS2+PM1+PM2+PM5+PP3). Considering that the proband’s mother had a similar mild phenotype (mild thickening of the skin on the soles of the hands and feet) and the result of WES (∼120×) was the heterozygous ratio of 2/83, it was speculated that the proband’s mother exhibited mosaicism for this variant. So, the proband’s mother was subjected to targeted ultra-high depth sequencing (∼20,000×), and the result showed that she really had low-level mosaicism with a variant allele fraction of 0.006%.

The *GJB2* gene (NM_004004.6) contains two exons; only exon 2 has a coding function, and most variants occur in exon 2, causing structural and functional abnormalities in Cx26 (NP_003995.2). Cx26 is a transmembrane protein that transits the membrane four times and is divided into five regions: N-terminal region (NT), transmembrane regions (TM1, TM2, TM3, and TM4), extracellular regions (EC1 and EC2), cytoplasmic region (CL), and C-terminal regions (CT). So far, most of the reported variants causing DFNB1A have been concentrated in the transmembrane regions, especially TM1 and TM2, and the variants causing DFNA3A mainly focus on the EC1, EC2, TM3, and TM4. Furthermore, the majority of SHL-causing variants have been concentrated in EC1 and EC2, with PPKDFN-causing variants mainly in EC1 and at the junction of EC1 and TM2. The variant site p.R75P in this study is located at the junction of EC1 and TM2 (Figures 1B,C).

**FIGURE 2:**
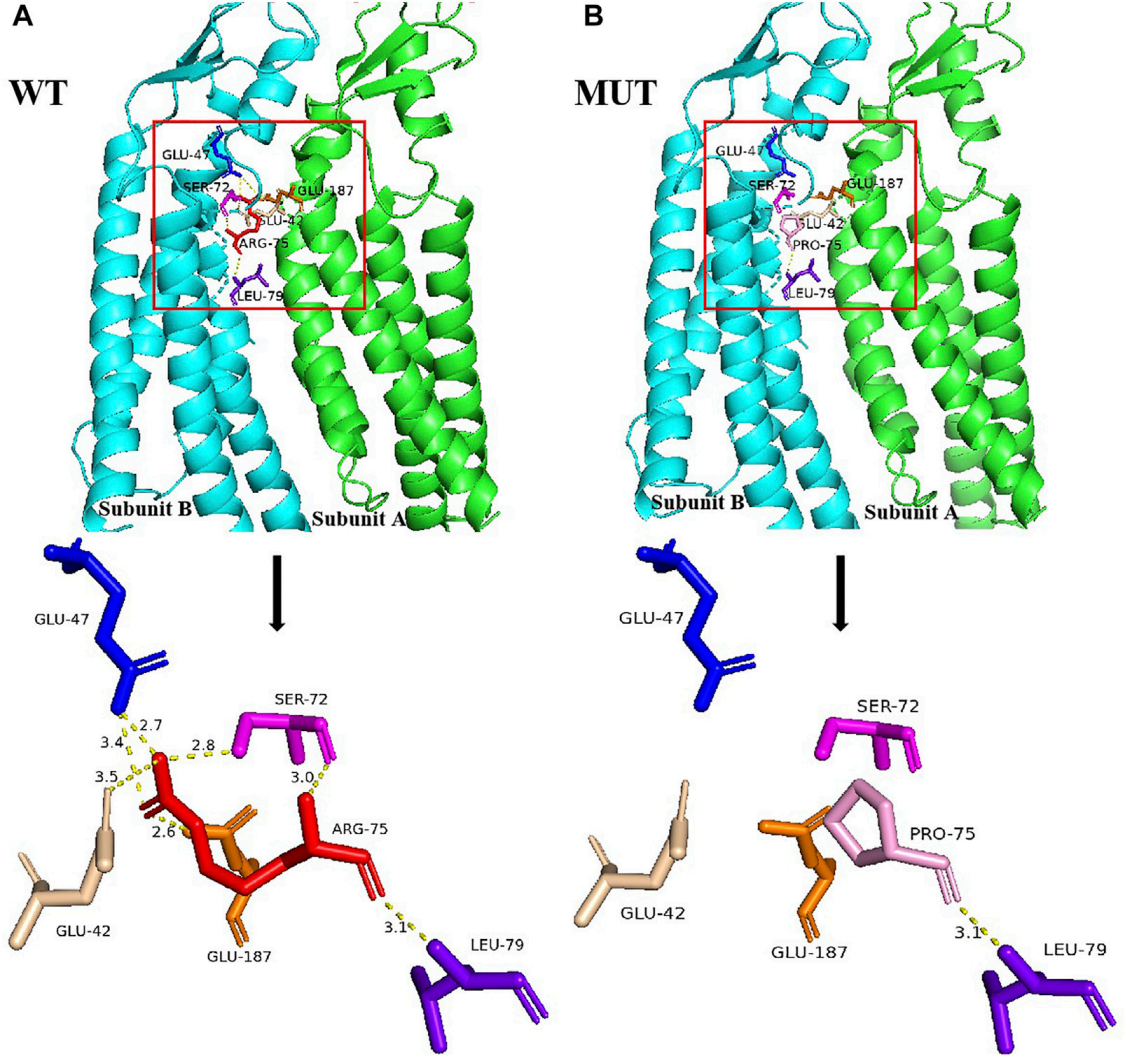
Structural analysis of the *GJB2* p.R75P variant. **(A)** Location of Arg75 and the nearby residues it interacts with, and the local spatial structure of the residues Arg75, Leu79, Ser72, and Glu47 in subunit B and Glu187 and Glu42 in subunit A. Hydrogen bonds are indicated as yellow dotted lines. Other subunits of Cx26 have been omitted for clarity. **(B)** Location of Pro75 and the nearby residues it interacts with, and the local spatial structure of the residues Pro75, Leu79, Ser72, and Glu47 in subunit B and Glu187 and Glu42 in subunit A. Hydrogen bonds are indicated as yellow dotted lines.

Structural analysis demonstrated that the Arg75 of subunit B was located at the edge and adjacent to subunit A. In wild-type Cx26, Arg75 interacted with Glu47, Ser72, and Leu79 in intra-subunit B as well as Glu42 and Glu187 in adjacent subunit A, to facilitate the assembly of hexamer linkers and enhance the structural stability of gap junction channels, through hydrogen bonding (Figure 2A). However, the spatial conformation was greatly changed when Arg75 was substituted with Pro75 in mutant Cx26, showing that Pro75 could not form hydrogen bonds with other amino acid residues in addition to Leu79 (Figure 2B). In addition, the change from a positively charged Arg to a neutral Pro may affect the regional environment, which could subsequently impair permeation properties of Cx26.

## Discussion and Conclusion

Variations in the *GJB2* gene can cause autosomal recessive and autosomal dominant deafness, and variants in *GJB2* are the most common cause of non-syndromic autosomal recessive sensorineural hearing loss; however, only a few variants that lead to dominant patterns of inheritance have been described worldwide. We report a female patient with PPKDFN caused by the heterozygous dominant variant c.224G>C in the *GJB2* gene, which was unreported previously.

The Cx26 protein includes two extracellular domains and three intracellular domains: the intracellular ends of NT and TM1 form a charged complex that acts as a voltage sensor, and there are Ca^+^-binding sites in the TM1 and TM2 regions; EC1 and EC2 are highly conserved, participating in the docking of extracellular hemichannels to establish a complete intercellular communication channel and determining the compatibility of gap junction channels with other connexin proteins, along with containing Ca^+^-binding sites in EC1; CL and CT are related to the gating of the pH value of the gap junction channel. The Cx26 protein forms linkers with other subunits, and the linkers fuse in adjacent cells to form gap junction channels. These channels are important pathways for intercellular electrolytes, second messengers, and metabolites, playing an important role in information transmission and material exchange. The six connexins oligomerize to form hollow hexamers, called linkers or hemichannels, which are then translocated to the cytoplasmic membrane, and the two linkers located on two adjacent cell membranes are docked through two extracellular domains to form hydrophilic low-resistance channel. Connexins enable the transport of molecules smaller than 1 kDa between cells, participate in the regulation of synergistic activities between cells, and are highly expressed in a variety of tissues, including the cochlea, hair follicles, sweat glands, and the epidermis of the palms and soles of the feet (Leybaert et al., 2017).

**FIGURE 3:**
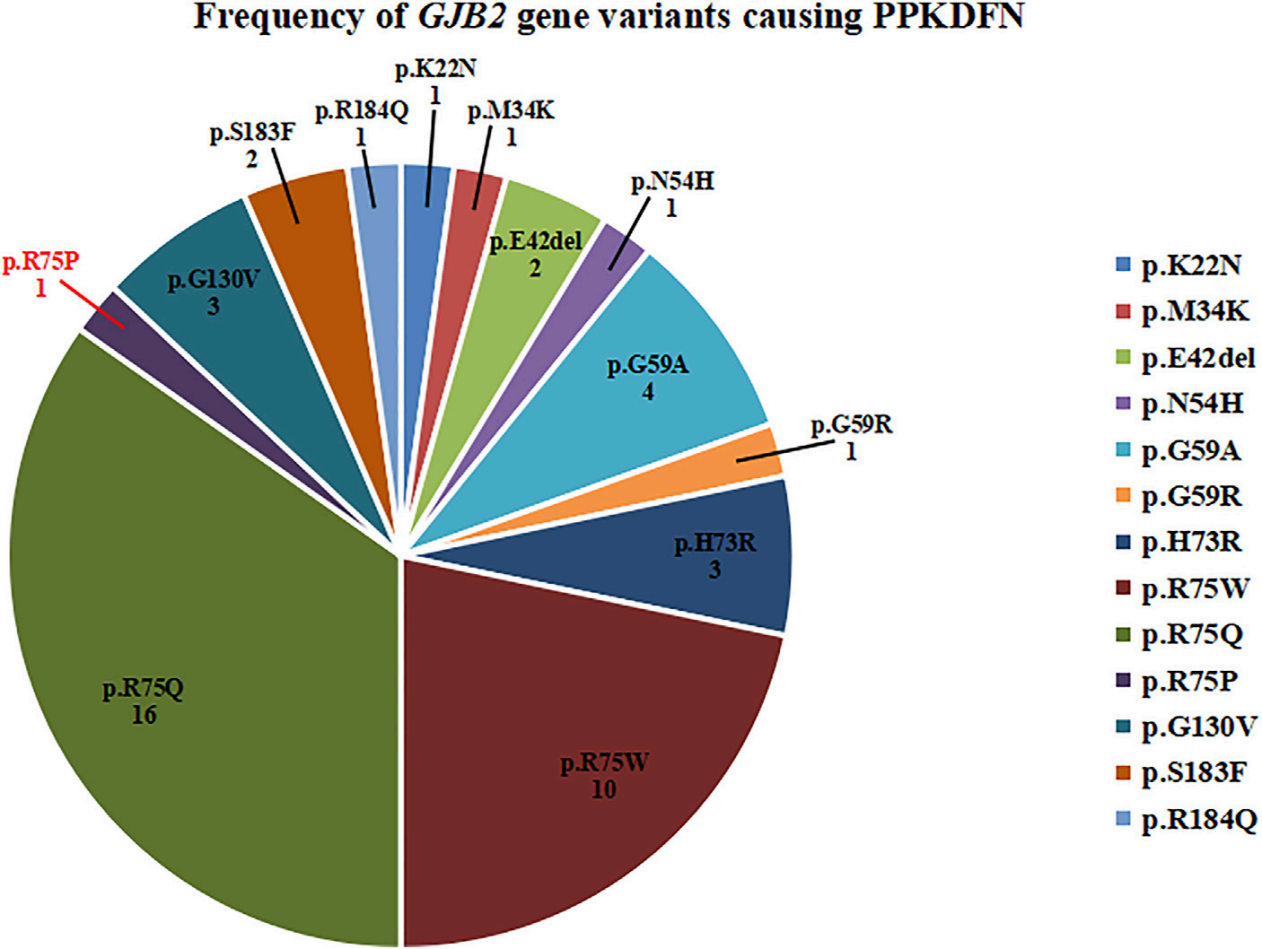
Frequency of *GJB2* gene variants causing PPKDFN. p.R75Q and p.R75W are hotspot variants of PPKDFN.

Variants in the TM1 and TM2 regions predispose to dysregulation of calcium homeostasis, resulting in impaired electrolyte exchange, which affects nerve signaling and leads to deafness. Therefore, TM1 and TM2 are variant hotspots for DFNB1A. Variants in the EC1 and EC2 regions can lead to structural defects in gap junction channels, which prevent the transport of small molecules such as electrolytes, metabolites, and second messengers between cells, resulting in multiple systemic diseases such as deafness, skin abnormalities, and ocular abnormalities. Thus, EC1 is considered a variant hotspot for SHL. The novel variant p.R75P is located at the junction of EC1 and TM2, leading to severe PPKDFN. Until now, the variant sites of PPKDFN are mainly concentrated on the EC1 region, which is consistent with the characteristics of SHL. Heterozygous variants in *GJB2* known to cause PPKDFN inhibit the formation of gap junction or leaky channels, cause defects in Cx26 transport to the cell membrane, and laterally interfere with the function of other connexin gap junction channels (Shuja et al., 2016). The most common variants causing PPKDFN are p.R75W and p.R75Q (Figure 3), while the hotspot variants that cause other SHL are p.D50N and p.D66H.

The p.R75 site is strongly conserved among all Cx26s in different species (Richard G et al., 1998). It can form hydrogen bonds with nearby amino acid residues and is involved in the interaction between subunits. Therefore, the p.R75 variant can lead to hydrogen-bonding defects, and the stability of Cx26-formed metabolites or ion exchange channels is destabilized, making the hair cells lose function during development causing hearing impairment, and they also form leaky channels that destabilize epidermal calcium homeostasis and lead to skin abnormalities (Cocozzelli and White, 2019; Mammano, 2019). At present, the pathogenic heterozygous variants found at this site are p.R75W and p.R75Q. In this study, a novel variant p.R75P was found, which can lead to hydrogen bond defects and further makes the structure and function of Cx26 and its polymerized gap junction channel abnormal.

PPKDFN is characterized by slow progression and manifested as palmoplantar keratoderma and hearing loss. Patients develop rough, thicker skin on the palms and soles (palmoplantar keratoderma) in childhood, with hearing loss ranging from mild to severe and worsening over time. Recent studies have shown that gene variants in PPKDFN patients are almost all missense variants in addition to p.E42del, and most of the deafness is congenital. PPKDFN has high phenotypic heterogeneity (Table 1). Evenly, it has been reported that the p.R75 variant only causes deafness and not skin lesions (Pavithra et al., 2015; Richard G et al., 1998), and even within the same family, the signs and symptoms of the disease may vary, with some individuals having only skin abnormalities while others only hearing loss. Therefore, the occurrence of palmar toe hyperkeratosis may also be related to other genetic backgrounds, such as gene interactions, epigenetic changes, environmental factors, and random penetrance changes. At present, other genes that can cause palmoplantar hyperkeratosis have also been found, such as *KRT1*, *KRT9,* and *LOR* genes. Then, what needs to be further studied is whether there are variants or polymorphisms of *KRT1*, *KRT9*, and *LOR* genes at the same time in patients with PPKDFN caused by the *GJB2* gene variant (Terron-Kwiatkowski A et al., 2002; Codispoti A et al., 2009). The simultaneous occurrence of these genes and *GJB2* gene variants can aggravate skin diseases. Therefore, the specific skin and eye abnormalities and degrees need to be combined with the interaction of other genes to synthesize judgment. Our patient had both delayed-onset deafness and severe palmoplantar hyperkeratosis, expanding the phenotypes spectrum of the *GJB2* variants. In addition, we have found other gene variants in the proband (Supplementary Table S1), *WFS1* gene c.2414G>A (p.R805Q) and *MORC2* gene c.2567G>T (p.C856F), which were inherited from the mother. Variants in the *WFS1* gene can cause hearing loss, which is rare. The protein–protein interaction exists between GJB2 and WFS1, and the interaction of these two gene variants may aggravate the degree of hearing loss in the proband (Khan et al., 2019; W. H.; Liu et al., 2019).

**TABLE 1:** Clinical phenotypes of PPKDFN patients with the variation reported in the *GJB2* gene.

Family	Patient	Relation to the proband	Age (years)	Variant	Hearing loss	Epidermal abnormality	Study
1	1–1	Proband	7	c.66G>T; p.K22N/-	Delayed-onset, profound	No	Stanghellini et al. (2017)
	1–2	Sister	23	c.66G>T; p.K22N/-	Delayed-onset, mild	No	
	1–3	Mother	46	c.66G>T; p.K22N/-	Delayed-onset, severe	Diffuse palmoplantar hyperkeratosis	
	1–4	Maternal grandfather	78	c.66G>T; p.K22N/-	Delayed-onset, severe	Diffuse palmoplantar hyperkeratosis	
2	2–1	Proband	25	c.101T>A; p.M34K/c.35delG; p.G12Vfs*2	Congenital, profound	PPK, chronic mucocutaneous candidiasis, and resorption of the finger tips; erythematous, flat, macular rash on bilateral legs, palmoplantar hyperkeratosis with fissuring, and dystrophy of 20 nails	Pang et al. (2014)
	2–2	Mother	N/A	c.101T>A; p.M34K (variant allele fraction of 17%)/-	No	No	
3	3–1	Proband	15	c.125delAGG; p.E42del/c.35delG; p.G12Vfs*2	Congenital, severe	Severe palmoplantar hyperkeratosis with deep fissures, shallow pits, and horizontal ridges of the nails	Rouan F et al. (2001)
	3–2	Father	41	c.125delAGG; p.E42del/-	Congenital	Mild diffuse hyperkeratosis of the sole with accentuated skin markings and marked callus formation over the pressure points	
4	4–1	Proband	12	c.160A>C; p.N54H/-	Congenital, profound	Severe palmoplantar hyperkeratosis and knuckle pads (fingers)	Akiyama et al. (2007)
5	5–1	Proband	N/A	c.176G>C; p.G59A/-	Congenital, profound	Severe palmoplantar hyperkeratosis	Heathcote K et al. (2000)
	5–2	Mother	N/A	c.176G>C; p.G59A/-	Congenital, profound	Severe palmoplantar hyperkeratosis	
	5–3	Aunt	N/A	c.176G>C; p.G59A/-	Congenital, profound	Severe palmoplantar hyperkeratosis	
	5–4	Maternal grandfather	N/A	c.176G>C; p.G59A/-	Congenital, profound	Severe palmoplantar hyperkeratosis	
6	6–1	Proband	8	c.175G>C; p.G59R/-	Congenital, profound	Striate linear palmoplantar hyperkeratosis and mild hyperkeratosis over one elbow and knuckle pads (fingers)	Leonard et al. (2005)
7	7–1	Proband	40	c.219A>G; p.H73R/-	N/A, severe	Severe palmoplantar hyperkeratosis, accentuation on the thenars, hypothenars, and the arch of the feet	de Zwart-Storm et al. (2008)
	7–2	Daughter	9	c.219A>G; p.H73R/-	Congenital, severe	Discrete, hyperkeratotic papules with accentuation of the palmar lines as well as larger hyperkeratosis on plantar areas affected by pressure	
	7–3	Son	2	c.219A>G; p.H73R/-	Congenital, profound	Mild keratotic papules of the same pattern	
8	8–1	Proband	17	c.223C>T; p.R75W/c.235delC	Congenital, profound	Severe peeling (soles) and thickening (palms and soles) of the skin, keratoderma (palms and feet), erythema (hands and feet), knuckle pads (fingers), and deep fissures (hand and feet); the epidermal abnormalities were extended to wrists, ankles, and the dorsal area of hands and feet	Pang et al. (2014)
	8–2	Mother	44	c.223C>T; p.R75W/-	Congenital, profound	Thickening of the skin (palms and soles), keratoderma (feet), erythema (hands and feet), knuckle pads (fingers), and callus (soles)	
9	9–1	Proband	5	c.223C>T; p.R75W/-	Congenital, profound	Thickening of the skin (palms), keratoderma (palms), and thickened (toes) or brittle nails (fingers)	Pang et al. (2014)
	9–2	Sibling	3	c.223C>T; p.R75W/-	Congenital, profound	Thickening of the skin (palms), minor peeling and erythema (palms), and brittle nails (fingers)	
10	10–1	Proband	3	c.223C>T; p.R75W/-	Congenital, severe	Diffuse palmoplantar hyperkeratosis and keratotic plaques on the knuckle areas	Lee et al. (2010)
	10–2	Mother	27	c.223C>T; p.R75W/c.79G>A; p.V27I	Congenital	Diffuse palmoplantar hyperkeratosis	
	10–3	Aunt	N/A	N/A	Congenital	Diffuse palmoplantar hyperkeratosis	
	10–4	Maternal grandfather	Passed away	N/A	Congenital	Diffuse palmoplantar hyperkeratosis	
11	11–1	Proband	N/A	c.223C>T; p.R75W/-	Congenital, profound	Diffuse palmoplantar hyperkeratosis	Richard et al. (1998)
	11–2	Father	N/A	c.223C>T; p.R75W/-	Congenital, profound	Diffuse palmoplantar hyperkeratosis	
12	12–1	Proband	17	c.224G>A; p.R75Q/c.109G>A; p.V37I	Congenital, profound	Thickening (palms and soles) and peeling (soles) of the skin, keratoderma (palms), erythema (hands and feet), knuckle pads, and circular keratotic constriction band (fingers)	Pang et al. (2014)
	12–2	Half sibling	26	c.224G>A; p.R75Q/-	Delayed-onset (∼10 years), mild	Minor thickening of the skin (palms only) and keratoderma (palms)	
	12–3	Mother	49	c.224G>A; p.R75Q/c.109G>A; p.V37I	Congenital, profound	Thickening (palms and soles) of the skin, keratoderma (palms), knuckle pads and circular keratotic constriction band (fingers), and callus (soles)	
13	13–1	Proband	3	c.224G>A; p.R75Q/-	Congenital, profound	Thickening and peeling of the skin (palms and soles) and erythema (hands and feet)	Pang et al. (2014)
14	14–1	Proband	31	c.224G>A; p.R75Q/-	Congenital, severe	Thickening of the skin (palms), keratoderma (palms), and knuckle pads (fingers)	Pang et al. (2014)
15	15–1	Proband	N/A	c.224G>A; p.R75Q/c.35delG; p.G12Vfs*2	Congenital, profound	Severe palmoplantar hyperkeratosis	Bousfiha et al. (2016)
	15–2	Brother	N/A	c.224G>A; p.R75Q/c.35delG; p.G12Vfs*2	Congenital, profound	Severe palmoplantar hyperkeratosis	
	15–3	Mother	N/A	c.224G>A; p.R75Q/-	Congenital, profound	Severe palmoplantar hyperkeratosis	
	15–4	Maternal grandfather	N/A	c.224G>A; p.R75Q/-	Congenital, profound	Severe palmoplantar hyperkeratosis	
16	16–1	Proband	5	c.224G>A; p.R75Q/-	Congenital, severe	Mild palmoplantar hyperkeratosis	Uyguner O et al. (2002)
	16–2	Sister	9	c.224G>A; p.R75Q/-	Delayed-onset, mild	Mild palmoplantar hyperkeratosis	
	16–3	Father	39	c.224G>A; p.R75Q/-	Delayed-onset, profound	Mild palmoplantar hyperkeratosis	
	16–4	Grandmother	N/A	c.224G>A; p.R75Q/-	Delayed-onset	Mild palmoplantar hyperkeratosis	
17	17–1	Proband	29	c.224G>A; p.R75Q/-	N/A, severe	Diffuse palmoplantar hyperkeratosis extending to the wrist and the dorsal surfaces of the feet	Jiang et al. (2014)
	17–2	Mother	54	c.224G>A; p.R75Q/-	N/A, profound	Diffuse palmoplantar hyperkeratosis extending to the wrist and the dorsal surfaces of the feet	
	17–3	Uncle	52	c.224G>A; p.R75Q/-	N/A, profound	Diffuse palmoplantar hyperkeratosis extending to the wrist and the dorsal surfaces of the feet	
18	18–1	Proband	24	c.224G>C; p.R75P/-	Delayed-onset, severe	Severe palmoplantar hyperkeratosis	This study
	18–2	Mother	48	c.224G>C; p.R75P (variant allele fraction of 0.006%)/-	NO	Mild keratoderma (palms and soles)	
19	19–1	Proband	3	c.389G>T; p.G130V/c.35delG; p.G12Vfs*2	Congenital, profound	Mild palmoplantar hyperkeratosis and mild ungual dystrophy	Iossa et al. (2009)
	19–2	Mother	N/A	c.389G>T; p.G130V/-	Delayed-onset, severe	Mild palmoplantar hyperkeratosis and mild ungual dystrophy	
	19–3	Maternal grandfather	N/A	c.389G>T; p.G130V/-	Delayed-onset, profound	N/A	
20	20–1	Proband	43	c.548C>T; p.S183F/-	N/A, profound	Severe keratoderma (palms and soles), knuckle pads (fingers), and flat, hyperkeratotic translucent plaques consisting of confluent hyperkeratotic papules	de Zwart-Storm et al. (2008)
	20–2	Daughter	5	c.548C>T; p.S183F/-	Diagnosed at 5 years, profound	Mild keratoderma (palms and soles)	
21	21–1	Proband	33	c.551G>A; p.R184Q/-	Congenital, severe	Thickening and peeling of the skin (soles), keratoderma (soles), callus (soles), thickened nails (fingers), and spoon nails (toes)	Pang et al. (2014)

Individuals that originate from a single fertilized egg but contain two cell lines with different genetic makeup are called mosaic. According to the period of variant and tissue distribution, mosaicism is classified into three types: somatic mosaicism, gonadal mosaicism, and both somatic and gonadal mosaicism (Spinner and Conlin, 2014). The latter two types of mosaic parents can pass the variant on to their offspring. Therefore, it is very important for family genetic counseling of genetic diseases to identify parental mosaicism early. Traditional Sanger sequencing is prone to human error due to its low resolution. At present, the detection methods of low-level mosaicism mainly include pyrosequencing, PCR product cloning and sequencing, allele-specific fluorescence quantitative PCR, denaturing high-performance liquid chromatography (dHPLC), high-depth sequencing, and RainDrop droplet digital PCR (ddPCR). Among these, high-deep sequencing and ddPCR are commonly used methods of mosaic detection in recent years, which have high sensitivity and can quantitatively analyze variants (Tone et al., 2014; Cohen et al., 2015). In this study, maternal mosaicism was discovered by 120× WES, and further targeted high-depth sequencing technology was used to verify maternal mosaicism. After targeted ultra-high depth sequencing of the patient’s mother, the result of the p.R75P variant percentage was 0.006%, indicating that she has a low-level mosaicism. Due to the extremely low variant level of the proband’s mother, she showed only mild symptoms, which is prone to missed diagnosis and indicating that the abnormal GJB2 protein expressed in this low proportion did not cause obvious clinical manifestations. Our study shows that the WES can give evidence of parental mosaicism and should be of concern to clinicians and genetic counselors. In addition to focusing on *de novo* variants, parental mosaicism should also be considered when clinically identifying pathogenic variants, which can provide a reliable basis for genetic counseling and fertility guidance. In this case, the proband’s parents should conduct prenatal genetic testing before giving birth again to reduce the risk of disease recurrence. The proband also was at risk of having a child with PPKDFN during pregnancy, so we recommend that she undergo prenatal diagnosis or PGT-M to avoid the birth of a PPKDFN child.

Taken together, our study expands the *GJB2* gene variant spectrum, indicates that it is feasible and effective for the diagnosis of PPKDFN and detection of low-level mosaic variant through WES and ultra-deep targeted sequencing, and manifests that early detection of variant mosaicism enables more accurate genetic counseling and prenatal diagnosis for families with genetic disease.

## Data Availability

The datasets for this article are not publicly available due to concerns regarding participant/patient anonymity. Requests to access the datasets should be directed to the corresponding author.
